# Differential Expression of miRNAs and Their Predicted Target Genes Indicates That Gene Expression in Atlantic Salmon Gill Is Post-Transcriptionally Regulated by miRNAs in the Parr-Smolt Transformation and Adaptation to Sea Water

**DOI:** 10.3390/ijms23158831

**Published:** 2022-08-08

**Authors:** Alice Shwe, Aleksei Krasnov, Tina Visnovska, Sigmund Ramberg, Tone-Kari K. Østbye, Rune Andreassen

**Affiliations:** 1 Department of Life Science and Health, Faculty of Health Sciences, OsloMet-Oslo Metropolitan University, 0167 Oslo, Norway; 2 Nofima (Norwegian Institute of Food, Fisheries and Aquaculture Research), 1430 Ås, Norway; 3 Bioinformatics Core Facility, Oslo University Hospital, 0372 Oslo, Norway

**Keywords:** smoltification, Atlantic salmon, gill, microRNA, microarray transcriptome, high-throughput sequencing, seawater adaptation

## Abstract

Smoltification (parr-smolt transformation) is a complex developmental process consisting of developmental changes that lead to remodeling of the Atlantic salmon gill. Here, the expression changes of miRNAs and mRNAs were studied by small-RNA sequencing and microarray analysis, respectively, to identify miRNAs and their predicted targets associated with smoltification and subsequent sea water adaptation (SWA). In total, 18 guide miRNAs were identified as differentially expressed (gDE miRNAs). Hierarchical clustering analysis of expression changes divided these into one cluster of 13 gDE miRNAs with decreasing expression during smoltification and SWA that included the miRNA-146, miRNA-30 and miRNA-7132 families. Another smaller cluster that showed increasing expression consisted of miR-101a-3p, miR-193b-5p, miR-499a-5p, miR-727a-3p and miR-8159-5p. The gDE miRNAs were predicted to target 747 of the genes (DE mRNAs), showing expression changes in the microarray analysis. The predicted targets included genes encoding NKA-subunits, aquaporin-subunits, cystic fibrosis transmembrane conductance regulator and the solute carrier family. Furthermore, the predicted target genes were enriched in biological processes associated with smoltification and SWA (e.g., immune system, reactive oxygen species, stress response and extracellular matrix organization). Collectively, the results indicate that remodeling of the gill involves the post-transcriptional regulation of gene expression by the characterized gDE miRNAs.

## 1. Introduction

Atlantic salmon (*Salmo salar*) are anadromous, which means that they live in both fresh water (FW) and sea water (SW) during their life cycle [1,2]. In preparation for the SW phase, while still in freshwater, the salmon undergo smoltification, a process that transforms a freshwater dwelling parr into a saltwater-adapted smolt [3]. Smoltification is a complex developmental process that consists of changes in fish physiology (e.g., increased osmoregulation and salinity tolerance), morphology (e.g., slimmer body, silvering skin color and darkened fin margins) and behavior (e.g., decreased territorial behavior and increased schooling) [2,4]. Smoltification-related changes are highly energy demanding and have been shown to suppress immune function, making fish more susceptible to pathogens and diseases [5,6]. Photoperiod and seasonal temperature fluctuations are primary environmental cues that initiate the smoltification of wild salmon, while in salmon production, smoltification is induced by the use of artificial photoperiods [7]. Photoperiod regulators, as well as temperature changes, stimulate the light-brain-pituitary axis and lead to increased levels of growth hormone (GH), cortisol and thyroid hormones [1]. GH and cortisol interact to control the hyper-osmoregulatory mechanism in the gill, gut and kidney and together stimulate an increase in salinity tolerance as well as changes in growth and metabolism [1,8].

Atlantic salmon is the major aquaculture species in Norway, and in 2019, there was a production of farmed salmon of USD 7 billion [9]. Despite being the most successful aquaculture species, mortality during the SW phase remains a challenge and in 2020 as many as 52 of 290 million Norwegian farmed salmon transferred to sea died prior to harvest [10]. The mortality rate has been observed to be higher during the first 3 months after seawater transfer (SWT) [11,12]. Suboptimal smoltification has been reported as a contributory factor to death shortly after SWT [10,13]. Disease during the SW phase was identified as another important contributor to losses prior to harvest [14].

The gill is a complex multifunctional organ that plays a central role in gas exchange, nitrogenous waste excretion, pH balance, and osmoregulation and ion balance [15,16]. To maintain blood ion and acid-base balance, the gill of freshwater (FW) acclimated fish actively transports Na^+^ and Cl^−^ from dilute freshwater into the blood to match the passive loss of these ions in the urine and body surface. In contrast, SW acclimated fish drink SW to counter osmotic loss of water and secrete excess Na^+^ and Cl^−^ across the gills through specialized cells called ionocytes, mitochondria-rich cells (MRC), or chloride cells [17,18,19]. Therefore, the transformation of the gill from an ion-absorbing epithelium to an ion-secreting epithelium is an essential part of smoltification. Remodeling of the gill is also associated with higher levels of Na^+^/K^+^ -ATPase (NKA) activity [20]. The use of different homologs of the NKA subunits likely facilitates a life in SW, as smoltification is associated with a change in the expression of such homologs. The *nkaα1b* subunit has, e.g., been reported to increase in expression in smolt [15,21,22,23]. NKA is a membrane protein that energizes osmotic and ionic regulation in FW and SW by providing electrochemical force for the active movement of ions into or out of the fish across the gill epithelium [15,24]. In FW parr, the gill epithelium is rich in MRCs that express *nkaα1a*, a NKA isoform associated with freshwater and ionic uptake. During smoltification, there is an increase in the number of *nkaα1b* MRCs [1] that also contain Na^+^ K^+^ 2Cl^−^ cotransporter (*cftr*) and the apical cystic fibrosis transmembrane conductance regulator (*nkcc*) [22,25]. The *nkaα1b* MRCs in FW smolts are likely to be inactive in freshwater, but rapidly initiate salt secretion after SW exposure [25]. The elevated level of *nkaα1b* MRCs in SW smolts is associated with salinity tolerance and ionic secretion [25]. Currently, the gill NKA activity that increases during smoltification [22,25,26] together with gill mRNA levels of *nkaα1a* and *nkaα1b* [22] showing decrease and increase, respectively, are utilized as indicators of smolt development in salmon production. In gills, increased levels of growth hormone (GH) and cortisol are suggested to stimulate salinity tolerance and NKA activity by promoting the formation of seawater MRC [27]. In addition, GH and cortisol interact to control epithelial transport activity. Prolactin, on the other hand, inhibits the formation of seawater MRCs and promotes the development of freshwater MRCs [15].

The gills are also the main mucosal surfaces and immune barriers where there are cellular components of the innate immune system (macrophages, mast cells, granulocytes, dendritic cells and antimicrobial peptides) and the adaptive immune system (B cells, T cells and the teleost immunoglobulins IgM and IgT) [28]. A previous study reported that the gill mucus cell population increased in number and varied in histochemical staining in response to increased salinity [29]. Studies of gene expression in the gill of rainbow trout challenged with *Renibacterium salmoninarum* [30] and Atlantic salmon challenged with amoebic gill disease (AGD) [31] reported that immune-related proteins were induced in response to these pathogen invasions. In addition, genes involved in stress responses and xenobiotic metabolism have been reported to be upregulated in the gill of Atlantic salmon in response to smoltification and SW adaptation [6].

MicroRNAs (miRNAs) are small single-stranded non-coding RNAs (~22 nucleotides in length) that are involved in gene expression at the post-transcriptional level [32,33]. miRNAs are transcribed into primary miRNAs (pri-miRNAs) that are processed further into precursor miRNAs (pre-miRNAs) and then into mature miRNA duplexes through a cascade of several nuclear and cytoplasmic enzymatic processing steps [32,34]. Finally, either the 5p or the 3p strands of the mature miRNA duplex are loaded into Argonaute (AGO), which interacts with other proteins to form the miRNA-induced silencing complex (miRISC). The strand that is incorporated into AGO (termed “guide miRNA”) directs miRISC to its target mRNA, most often by interacting with the 3′ UTR of the target mRNAs. This interaction induces translational repression or degradation of the target mRNAs [35]. Several studies have demonstrated that one of the mature miRNAs is commonly present in a much higher abundance than the other mature miRNAs processed from the same precursor [36,37]. The highly abundant mature miRNA is assumed to be the biologically important mature miRNA incorporated into AGO (referred to as guide miRNA), while the other lowly abundant miRNA is assumed to be the passenger miRNA that is quickly degraded when not incorporated into AGO [38,39].

The miRNAs in Atlantic salmon have been well characterized [36]. A recent study has also provided a transcriptome of more than 77,000 full-length (FL) sequenced mRNA transcripts using an FL-error-corrected mRNA sequencing approach [40]. This transcriptome includes the different splice variants expressed in the gills of Atlantic salmon during smoltification. The 3′UTRome from this FL sequenced transcriptome has been analyzed for its potential as miRNA targets, and a comprehensive searchable database of predicted mRNA targets for any of the mature miRNAs in Atlantic salmon was recently made publicly available (https://github.com/AndreassenLab/MicroSalmon/, accessed on 10 May 2022) [41].

MicroRNAs act as vital regulators of many developmental processes in vertebrates [42,43]. The study of miRNA expression in the gills of zebrafish treated with *Staphylococcus aureus* has, e.g., shown that miRNAs are involved in regulation of innate immune processes, apoptosis, defense responses and antibacterial responses [44]. Similarly, there are several challenge studies in Atlantic salmon showing that groups of miRNAs respond to virus diseases [45,46,47]. In addition, miRNAs have been reported to change their expression in the gills of Atlantic salmon in response to salinity challenges [48]. Our recent studies conducted in the head kidney [49] and liver [50] of Atlantic salmon identified a subset of miRNAs that were differentially expressed (DE) in response to smoltification and SW adaptation. The following enrichment analysis of the predicted targets of these DE miRNAs indicated that they were enriched in the biological processes associated with smoltification and SW adaptation in each of these organs [49,50].

Gill is an important organ involved in radical remodeling of its function during smoltification to cope with the osmotic and immunological challenges of SW [16,51,52], and miRNAs may play an important part in this reorganization of the gill functional system. The present work therefore aims to characterize DE miRNAs and their potential interactions in gene regulatory networks associated with smoltification and SW adaptation. Together, this study may provide a more extensive understanding of miRNA’s role in fine-tuning gene expression associated with smoltification-driven changes in the gills.

## 2. Results

### 2.1. Total RNA Extraction, Library Preparation and Small RNA Sequencing

Total RNA from 42 gill samples was successfully extracted, showing RNA concentrations that ranged from 301 to 2234 ng/µL and RIN values above 8. The quality measurements (Table S1) showed that all samples were fit for small RNA sequencing and microarray analyses. Subsequently, individual small RNA libraries were successfully generated and sequenced. The total number of raw reads ranged from 9.2 to 23 million. After adapter trimming and size filtering of raw reads, the total number of clean reads ranged from 5.9 to 15 million. The percentage of clean reads uniquely mapped as *Salmo salar* mature miRNAs from each sample ranged from 79.2 to 82.8. All raw sequenced files were submitted to NCBI with the BioProject accession number PRJNA681317. An overview of all samples, including their RNA concentration, RIN, number of raw reads, number of trimmed and size filtered reads, reads uniquely mapped as mature miRNAs and the SRA accession numbers are given in Table S1.

### 2.2. miRNAs Differentially Expressed in Atlantic Salmon Gill during Smoltification and SW Adaptation

To identify miRNAs that were differentially expressed during the smoltification and SW adaptation periods, expression analysis of small RNA sequencing data was conducted by comparing T1 (parr, one day prior to light treatment) to each of the other five sampling points: T2–T3 (the ongoing smoltification period), T4 (post-smoltification) and T5–T6 (post-SWT). In total, 32 miRNAs belonging to 22 miRNA families were identified as differentially expressed in at least one of the five time points compared. The mature sequences of the DE miRNAs and their relative expression changes at each time point (T2–T6) are provided in Table S2.

**Figure 1:**
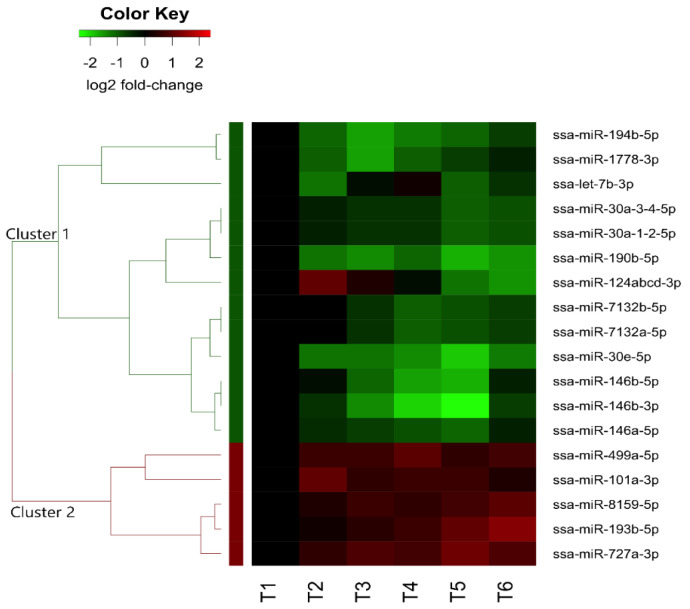
A heatmap illustrating the results from hierarchical clustering analysis of the 18 guide DE miRNAs. Each row represents a miRNA, and each column represents the expression changes at each time point relative to T1 (pre-smolt). T2-T4 and T5-T6 show the relative expression changes during the smoltification period and post-SWT period, respectively. The dendrogram and the row side colors on the left show the two major clusters of gDE miRNAs (Cluster 1-green, Cluster 2-red). The direction of expression changes (log2 fold-change) is illustrated by the color key above the heatmap.

Among the 32 DE miRNAs, 18 were denoted as guide DE miRNAs (gDE miRNAs), as they were the highly abundant mature miRNAs (10 times higher) of the two miRNAs derived from the same precursors (see methods, Section 4.5). The read count data used to annotate the gDE miRNAs are given in Table S3. Hierarchical analysis of the expression changes of the gDE miRNAs revealed one major cluster with decreased expression (Cluster 1) and one major cluster with increased expression (Cluster 2) over the time points compared. A heatmap illustrating the changes of the 18 gDE miRNAs is shown in Figure 1. A similar heatmap including all 32 DE-miRNAs is given in Supplementary Figure S1.

Cluster 1 consisted of 13 gDE miRNAs belonging to 8 different miRNA families that all showed a decrease in their expression (Figure 1 and Table 1). The majority of the gDE miRNAs in cluster 1 were significantly downregulated in smoltified fish (T4) and one week post-SWT (T5). Although ssa-miR-1778-3p and the ssa-miR-7132b family were significantly downregulated in smoltified fish (T4), the decrease was not significant post-SWT (T5 and T6).

Cluster 2 consisted of five gDE miRNAs (Figure 1 and Table 2) from five different miRNA families that all showed general increases that were significant at various time points. Three of the gDE miRNAs, ssa-miR-193b-5p, ssa-miR-727a-3p, and ssa-miR-8159-5p, were significantly upregulated post-SWT (T5 and T6). While ssa-miR-101a-3p was significantly upregulated at T2, ssa-miR-499a-5p was significantly upregulated in the smoltified fish (T4). As expected, the gDE miRNAs belonging to the same families were clustered together and revealed very similar expression changes (Table 1).

### 2.3. Identification of DE mRNAs Associated with Smoltification and SW Adaptation

Microarray analysis was conducted on the same gill samples and time points as used for miRNA expression analysis to identify mRNAs differentially expressed in response to smoltification and SW adaptation (see Methods, Section 4.6). The analysis of the 44K Salgeno-2 microarray revealed 2382 mRNAs that were significantly different expressed. The expression changes of DE-mRNAs, along with their *p*-values, are provided in Table S4.

An additional gene enrichment analysis was carried out to reveal the particular biological processes and gene pathways associated with the DE-mRNAs. Several immune-related biological processes, including adaptive immune response, cytokine-mediated signaling, leukocyte migration, inflammatory response, antigen processing and presentation, and platelet degranulation, were significantly enriched. More general gene ontology groups, such as cell process, development, metabolism, structure and tissue, were also enriched in agreement with the developmental transformation occurring during smoltification. Similarly, the KEGG gene pathway enrichment analysis showed that immune-related pathways, such as cytokine–cytokine receptor interaction and the Jak–STAT signaling pathway, were enriched. Also, the enrichment of gene pathways associated with metabolism (e.g., steroid hormone biosynthesis and aldosterone-regulated sodium reabsorption), structure (phagosome) and tissue (tight junction) were in accordance with the findings from enrichment analysis of biological processes. An overview of all enriched biological categories and gene pathways revealed in the analysis of DE mRNAs is given in Table S5.

Notably, several genes that were previously reported to be associated with developmental changes that were decisive for good or poor smolt quality and regularly used as biomarkers of smolt success were among the DE mRNAs. They all revealed changes in expression, as expected, for successful smoltification. Some of these genes were Cytochrome P450 1A1 (*cyp1a1*) [53], intracellular protease calpain 9 (*capn9*) [54], calcium depot protein S100-A1 (*s100a1*) [55] and several of the genes encoding NKA α- and β-subunits [1,22,26,56]. Also, the Na^+^ K^+^ 2Cl^−^ cotransporter (*nkcc*) and the apical cystic fibrosis transmembrane conductance regulator (*cftr*), which are major ion transport proteins present in gill epithelium, revealed increased expression during smolt development in agreement with changes associated with SW acclimation [22].

### 2.4. Prediction of Target Genes of the gDE miRNAs

To better understand the role of gDE miRNAs in the regulation of gene expression, we performed in silico target prediction with 18 gDE miRNAs against the 2382 DE-mRNAs identified in the microarray analysis. The prediction analysis revealed 747 genes as putative targets. Furthermore, the full-length (FL) resource [41] (https://github.com/AndreassenLab/MicroSalmon/, accessed on 26 May 2022) showed that 2497 unique splice variants were transcribed from these 747 genes (or closely related paralogs). Table S6 provides a complete overview of the DE mRNA targets along with their targeting gDE miRNAs, gene names and symbols including transcript accession numbers and gene ontology (GO) terms.

**Figure 2:**
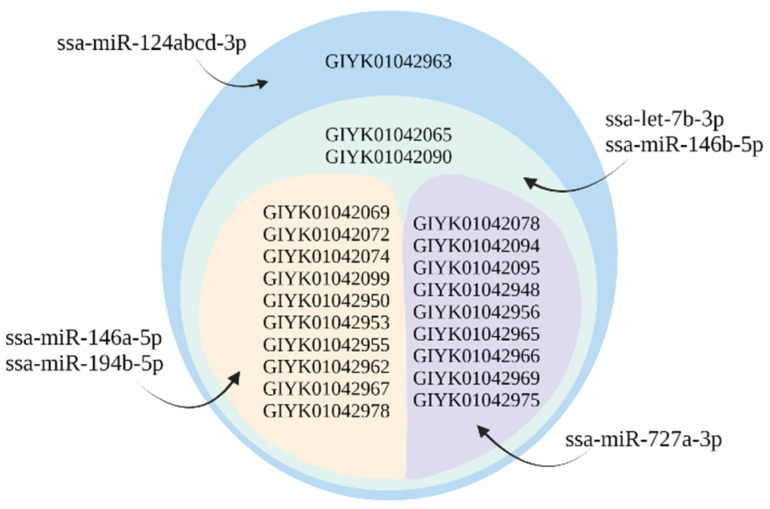
Six gDE miRNAs targeting particular splice variants of *nkaα1b* (22 isoforms). Ssa-miR-124abcd-3p was predicted to target all the splice variants shown in all the circular layers, while ssa-let-7b-3p and ssa-miR-146b-5p targeted transcripts belonging to the second and inner layers. Ssa-miR-146a-5p and ssa-miR-194b-5p targeted only the splice variants in the left inner layer, while ssa-miR-727a-3p targeted only the transcripts in the right inner layer.

**Figure 3:**
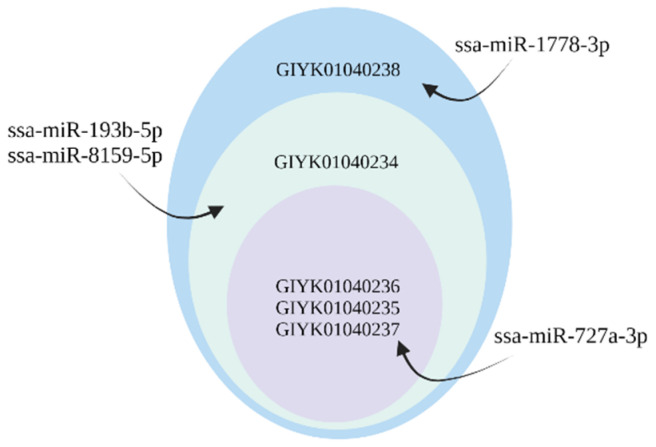
Four gDE miRNAs targeting particular splice variants of *cftr1* (five isoforms). Ssa-miR-1778-3p was predicted to target all the splice variants shown in all the circular layers, while ssa-miR-193b-5p and ssa-miR-8159-5p targeted transcripts belonging to the second and inner layers. Ssa-miR-727a-3p targeted only the transcripts in the inner layer.

Among the predicted target genes was *cyp1a1*, a gene used as a biomarker for exposure of contaminants in teleost fish [53]. All seven isoforms of this gene were targeted by ssa-miR-1778-3p (Table 3). In addition, the NKA transporting subunit α isoforms (*α1a* and *α1b*) which have been used as biomarkers to monitor smoltification [22] and the NKAβ subunit were miRNA targets. Two gDE miRNAs targeted the four different *nkaα1a* isoforms (Table 3), while six gDE miRNAs targeted the different splice variants (isoforms) of *nkaα1b* (Figure 2). The gDE miRNAs revealed a difference in which of the *nkaα1a* and *nkaα1b* transcripts they targeted, as shown in Table 3 and Figure 2. All transcript variants of *nkaβ*, on the other hand, were targeted by the same three miRNAs ssa-miR-146a-5p, ssa-miR-146b-5p and ssa-miR-1778-3p. The transcript variants encoding *cftr1*, a major ion transport protein involved in salt secretion, were, such as NKA subunits, targeted differently by four gDE miRNAs (Figure 3). Other target genes serving important gill functions were the aquaporin subunit (*aqp11*) targeted by ssa-miR-499a-5p and the V-type proton ATPase subunit (*atp6v1e2*) targeted by ssa-miR-194b-5p, ssa-miR-499a-5p and the miR-146 family. The immune genes *flt3* [57] and *itgae* were targets of ssa-miR-727a-3p, while *cd209* [58] were targets of the 7132 family and ssa-miR-146b-3p. All are known to be expressed in dendritic cell-like clusters in gills [16]. Finally, there were 27 genes encoding components of the solute carrier family of membrane transporters (SLC) among the target genes. These membrane transporters facilitate the transport of amino acids that may serve to regulate cellular osmolality against hyperosmotic stress [59].

Notably, mRNA-miRNA expression of the *nkaα1a* isoforms and ssa-miR-8159-5p showed a negative correlation that was statistically significant (Spearman’s rho = −1 and *p* < 0.01). However, the negative correlation between the one *nkaα1a* isoform and ssa-miR-499a-5p was not statistically significant. The predicted targets of *nkaα1b* isoforms and ssa-miR-146a-5p and ssa-miR-146b-5p also showed negative correlations in expression that were statistically significant. On the other hand, the rest of the gDE miRNAs targeting *nkaα1b* showed both positive and negative non-significant correlations. There was no significant correlation between *cyp1a1* and its targeting ssa-miR-1778-3p or between *cftr1* and its targeting gDE miRNAs.

### 2.5. Enriched Biological Processes and Pathways Associated with Predicted Target Genes

**Figure 4:**
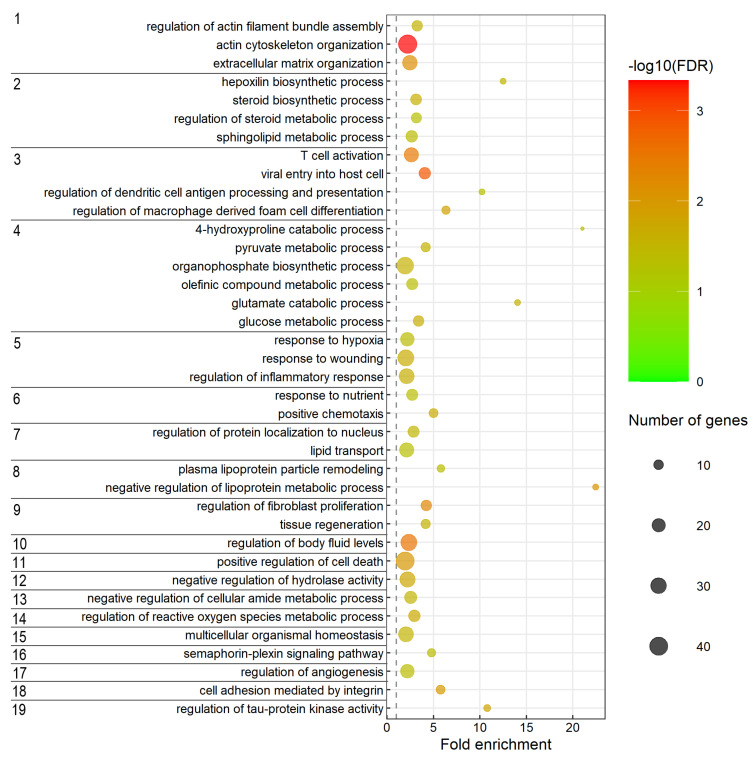
Biological processes significantly enriched in the predicted target genes of the gDE-miRNA dataset. The specific and representative subclasses of biological processes are shown on the y-axis. The enriched biological processes were grouped into the following categories: 1. Cellular organization, 2. Lipid metabolic process, 3. Immune system, 4. Organic substance metabolic process, 5. Response to stress, 6. Response to chemical, 7. Transport, 8. Protein metabolic process, 9. Regulation of cell growth, 10. Regulation of body fluid levels, 11. Regulation of cell death, 12. Regulation of molecular function, 13. Cellular nitrogen compound metabolic process, 14. Reactive oxygen species metabolic process, 15. Homeostatic process, 16. Signal transduction, 17. Angiogenesis, 18. Cell adhesion and 19. Phosphorylation.

Overrepresentation analysis (ORA) (see methods) was conducted to explore whether any of the predicted target genes were associated with particular biological processes and pathways relevant to smoltification and seawater adaptation. The specific subclasses of enriched biological processes (GO terms) are shown in Figure 4, while the enriched pathways are shown in Table 4. The complete output of the biological process enrichment analysis is given in Table S7.

Interestingly, several immune-related biological processes (e.g., regulation of macrophage-derived foam cell differentiation, regulation of dendritic cell antigen processing and presentation, viral entry into host cells, and T cell activation) (category 3 in Figure 4) were enriched. Expression changes of genes belonging to these immune category processes during smoltification were not unexpected, as gills are the main mucosal surfaces and immune barriers [28], and the gill mucus cell population increases in number in response to increased salinity [29]. Here, the ORA of the enriched predicted targets also indicated that these processes are regulated by miRNAs.

Biological processes associated with GH and cortisol, such as regulation of cell growth and steroid biosynthesis (category 2 and 9 in Figure 4), were also among the enriched targets. GH and cortisol are signaling agents that have previously been suggested to stimulate salinity tolerance and NKA activity by promoting the formation of seawater MRC [15]. The enrichment of genes involved in stress responses has been reported to be upregulated in the gill of Atlantic salmon in response to smoltification and SW adaptation [6]. The ORA suggested that such genes were also among those regulated by gDE miRNAs.

The ORA of enriched gene pathways (Table 4) did, in general, reveal gene pathways involved in the same biological processes as those that were enriched in the ORA of GO terms (Figure 4). Such enriched pathways were those associated with the immune system (e.g., Cytokine signaling pathway, innate and adaptive immune system). Extracellular matrix organization, signal transduction, and metabolism of lipids, vitamins and cofactors were other enriched pathways (Table 4).

## 3. Discussion

### 3.1. In Silico Analysis Revealed That Smoltification Biomarkers and Smoltification Key Genes Were among Predicted Targets of the gDE miRNAs

The miRNA expression analysis conducted here identified 32 miRNAs that were differentially expressed. All DE miRNAs from the same miRNA families showed similar expression changes. This observation is in agreement with previous studies reporting that miRNAs from the same family often share similar expression profiles and regulatory functions [60]. The fact that family members share seed sequences and otherwise are very similar also results in miRNAs targeting the same mRNAs [41]. Target gene analysis commonly predicts thousands of targets for each mature miRNA, some that obviously are not true targets if using passenger miRNAs in such analysis [38,39,41,47], and to optimize prediction analysis and the following enrichment analysis, the subset of the DE miRNAs used for these purposes were therefore the 18 gDE miRNAs. Although the differentiation between guide and passenger mature miRNAs was made based on their abundance (Table S3), we note that almost all denoted as gDE miRNAs have mature sequences, starting with a U in the 5′ end. This observation is in agreement with the preferences for which mature miRNAs are selected as guide miRNAs by Argonaute [39] and gives additional confidence that our annotation of gDE miRNAs is correct.

Seven of the gDE miRNAs revealed in this study were also reported to respond to smoltification and SWA in the liver [50]. Three of these, as well as ssa-miR-194b-5p, responded to smoltification and SWA in the head kidney (HK) [49] (Table 5). One of these (ssa-let-7b-3p) that were DE miRNAs in all three organs showed very similar expression patterns across the organs. Likewise, ssa-miRNA-101 and all members of the miRNA-30 family showed similar expression patterns in both the gill and liver. These similar dynamic changes across organs could indicate that they regulate some common general changes in cell homeostasis not related to organ-specific functions.

In contrast, ssa-miR-146a-5p and ssa-miR-146b-5p showed a decreasing expression pattern in the gill but an increasing one in the HK [49] and liver [50]. This could indicate that they have different regulatory functions in the gill than in the HK and liver. Similarly, 10 gDE miRNAs (Table 5) were differentially expressed only in the gill, not in HK or liver (for simplicity termed “gill-specific”). This observation supported that they, similar to the miR-146 family, are involved in the regulation of biological processes associated with gill-specific functions (e.g., nitrogenous waste excretion, osmoregulation and ion balance) [15,21]. One of these miRNAs, miR-124-3p, showed similar expression changes in the gill of saltwater adapting eel (Anguilla marmorata) [61] indicating these gill specific regulatory functions associated with SWA are conserved in teleost. Another gill-specific miRNA, ssa-miR-727a-3p, that showed a large increase at T5, targeted *flt3* and *itgae,* expressed in dendritic cell-like clusters in the gill, both showing large decreases in expression at T5 in this study.

The biological roles of gDE miRNA were further explored in the target prediction analysis. Such an analysis of potential miRNA target genes, as conducted in this study, is essential for elucidating their biological functions. miRNA prediction analysis was applied using all the different FL transcripts [40] transcribed from the differentially expressed genes revealed in the microarray analysis. Aiming to reduce the prediction of false target mRNAs, only target mRNAs predicted by the RNA hybrid and at least two of the other prediction tools (PITA, miRanda, TargetSpy) were included in the final putative target genes [41]. These results revealed that some of the gill-specific gDE miRNAs (Table 5) were predicted to target NKA isoforms, such as *nkaα1a* and *nkaα1b* and ion channel *cftr*. These genes are well known as molecular markers of smoltification [22,26]. Likewise, cytochrome P450 1A1 (*cyp1a1*), which is the most commonly used marker of xenobiotic metabolism [53] was targeted by ssa-miR-1778-3p, while aquaporin subunit (*aqp11*) was targeted by ssa-miR-499a-5p, both being gDE miRNAs only in the gill. Some targets of the gill-specific gDE miRNAs were thus well-known genes involved in smoltification. Consequently, these miRNAs may have essential gene regulatory roles in smoltification and SW adaptation.

The microarray analysis revealed that *nkaα1a* decreased expression during smoltification and after SWT, which is in agreement with previous studies [22,26]. Interestingly, the negative correlation in expression between ssa-miR-8159-5p and *nkaα1a* was significant (Spearman’s rho = −1, *p* = 1 × 10^−3^). This is as expected if *nkaα1a* is the target of ssa-miR-8159-5p and this regulation leads to post-transcriptional degradation of *nkaα1a* mRNAs. The expression of *nkaα1b* increased significantly during smoltification, which is also in agreement with previous studies [22]. Again, the four targeting gDE miRNAs ssa-miR-146a-5p, ssa-miR-124abcd-3p, ssa-miR-194b-5p and ssa-miR-146b-5p were negatively correlated. However, only the correlation between *nkaα1b* and ssa-miR-146a-5p and ssa-miR-146b-5p was significant (Spearman’s rho equal to −0.9 and −1.0, respectively). Nevertheless, these findings were also as expected, given that the miRNA–mRNA interactions result in degradation of mRNAs.

On the other hand, *cyp1a1* and *cftr1*, which were predicted to be targets of several gDE miRNAs, did not reveal any such significant negative correlations between the miRNAs and their predicted targets. However, it is unclear whether the target transcripts are always subjected to cleavage followed by degradation or whether the major mechanism of negative regulation is translational repression in Atlantic salmon. If certain target mRNAs are only translationally repressed [62], a negative correlation would not be expected. Clarifying whether target mRNAs are translationally repressed rather than degraded would be an important task in future studies to fully understand miRNA-guided post-transcriptional regulation in Atlantic salmon and teleosts. Such knowledge would also aid in identifying a true target mRNA and its targeting miRNA in expression studies similar to this one.

### 3.2. gDE miRNA Target Gene Enrichment Analysis

Enrichment analysis of differentially expressed target mRNAs was utilized to further explore the biological processes and pathways likely to be controlled by miRNA-guided post-transcriptional regulation. Interestingly, several biological processes associated with the immune system were enriched among the target genes. T-cell activation and regulation of dendritic cell antigen processing and presentation were enriched. Moreover, the regulation of macrophage-derived foam cell differentiation was also enriched. Together, this indicates that gDE miRNAs may regulate the well-described immune system and gill mucus cell population changes [28,29,63] during smoltification and SW adaptation.

The metabolism of organic acid and organic substances was also enriched among the target genes. The metabolic processes of pyruvate, organophosphate, olefinic compound and glucose including catabolic 4-hydroxyproline and glutamate, were included in this group. Such metabolites, especially pyruvate and glucose, are metabolized to provide energy essential for remodeling of the gill during this developmental transition [64]. The ORA of the targets indicates that some gDE miRNAs are involved in the regulation of increased energy production. Enrichment of biological process categories, such as regulation of actin filament bundle assembly and organization of actin cytoskeleton and extracellular matrix, also indicates that gDE miRNAs participate in the essential remodeling of the gill cell population. The gill membrane content of cholesterol and sulfatides increases during smoltification, which is suggested to be a pre-adaptation of salmon gills for salt secretion [65]. Interestingly, several biological processes associated with lipid metabolism were enriched in the target genes. Consequently, the gDE miRNAs may also be involved in regulating changes in the gill membrane lipid composition associated with SWA.

The findings from ORA indicate that gDE miRNAs are involved in a regulatory network that contributes to remodeling of the gill from an ion-absorbing epithelium to an ion-secreting epithelium during smoltification. Several of the predicted target genes were also among the essential key genes known to change their expression during this developmental transition. Collectively, the findings here indicate that miRNAs are important post-transcriptional regulators of parr-smolt transformation and adaptation to seawater.

## 4. Materials and Methods

### 4.1. Animal Welfare Statement

The experiment was conducted as part of the routine smolt production at the Nofima’s Research Station for Sustainable Aquaculture (Sunndalsøra, Norway). The maintenance of stock animals for experiments was in accordance with the Guidelines of the EU-legislation (2010/63/EU) as well as the Norwegian legislation on animal experimentation and was approved by the Norwegian Animal Research Authority. The experimental fish were not subjected to any pain or distress, and they were killed solely for the use of their tissues in this experiment. Thus, approval from the Norwegian Food Safety Authority was not required. 

### 4.2. Fish Holding and Sample Collection

A total of 70 Atlantic salmon from the Salmon Breed Model SB-Optimal were used for this study. The experimental fish selected here were the same fish as previously described by Shwe et al. [49,50]. The fish were housed in a one tank supplied with running water and they were fed commercial dry feed (Skretting, Norway). The fish were initially reared at continuous light (Light: Dark, LD 24:0) from the start of feeding. To introduce the winter signal for the initiation of smoltification, the light regime was then adjusted by decreasing daylight from 24 h to 12 h (LD 12:12) for 47 days, followed by 24 h daylight (LD 24:0) for 34 days (Table 6).

The seawater challenge test was performed once a week in the last 3 weeks before SWT using a salinity of 35‰. Seawater challenge test, blood plasma ions level (Cl^–^, Na^+^ and Mg^2+^) and change to silvery skin color indicated that the fish were fully smoltified 81 days after onset of photoperiod and temperature manipulation. Smolts with an average weight of 72.4 ± 8.7 g were then transferred directly to the seawater. The average weights at one week post-SWT and one month post-smolt were 63.2 ± 8.5 and 98.4 ± 14.9 g, respectively (Table 7). No mortality was observed during smoltification or after the SWT period. The management of smoltification used here is the same as that applied in today’s smolt production.

During the experimental period, the gill samples were collected at six time points (Table 7), T1: parr, 1 day prior to light treatment, T2: halfway through light treatment at the change from (LD 24:0) to (LD 12:12) and temperature to 12 °C (47 days post-onset of light treatment (POL)), T3: three-quarters into the light treatment period (67 days POL), T4: smolt, 1 days prior to SWT (81 days POL), T5: 1 week after SWT (88 days POL), and T6: 1 month after SWT (111 days POL). A summary of the experimental conditions is given in Table 7. At each sampling point, 10 fish were euthanized with an overdose of anesthetic metacain (MS-222; 0.1 g/L) and killed by a blow to the head prior to weighing and sampling. Gill samples were collected and frozen immediately in liquid hydrogen and stored at −80 °C until further processing.

### 4.3. Total RNA Extraction

Total RNA was extracted using the mirVanaTM miRNA Isolation Kit (Ambion, Life-Technologies, Carlsbad, CA, USA) according to the manufacturer’s protocol. The extracted RNA concentration and purity were assessed using a NanoDropTM1000 Spectrophotometer (Nanodrop ND-1000, Thermo Fisher Scientific, Wilmington, DE, USA). The integrity of the total RNA (RIN value) was measured using an Agilent 2100 Bioanalyzer in combination with an Agilent 6000 Nano Chip (Agilent Technologies, Santa Clara, CA, USA).

### 4.4. High-Throughput Sequencing of Mature miRNAs

Total RNA with A_260_:A_280_ ratio ranging from 2–2.2 and RIN ≥ 8 from seven gill samples from each time point (n = 42) was individually sequenced. A library of mature miRNAs was prepared using a QIAseq miRNA library kit (QIAGEN, Germantown, MD, USA) according to the manufacturer’s protocol. Next-generation sequencing (NGS) was performed using NextSeq 500 from Illumina (Illumina, Inc., San Diego, CA, USA). Both library preparation and sequencing were performed at the Norwegian Sequencing Centre (NSC; Oslo, Norway). All high throughput sequencing (HTS) samples have been submitted to the NCBI Sequence Read Archive Centre (SRA) (https://www.ncbi.nlm.nih.gov/sra, accessed on 4 May 2022) with accession bioproject number PRJNA681317. They will be automatically released by the NCBI at the publication of this study.

### 4.5. Processing of HTS Data and miRNA Expression Analysis

The quality of the raw sequencing data was controlled with FASTQC (v.0.11.8) (https://www.bioinformatics.babraham.ac.uk/projects/fastqc/, accessed on 5 May 2022). The adapter (5′ AACTGTAGGCACCATCAAT 3′) was trimmed followed by size filtering to discard all reads shorter than 18 bp and longer than 25 bp using Cutadapt (v.2.10) [66]. The filtered reads were quality checked with FASTQC to ensure that the final data set of clean reads was of good quality.

MirDeep2 analysis (v.2.0.0.7) [67] was performed on two gill samples from smoltified fish (T4) and two gill samples from saltwater-adapted fish for miRNA discovery, as described by Woldemariam et al. [36]. The analysis revealed no new miRNAs other than those already described in the Atlantic salmon miRNAome [36]. The Atlantic salmon miRNAome was therefore used as a reference index in the following differential expression analysis. Clean reads were aligned to the Atlantic salmon miRNAome [36] using STAR aligner software (v.2.5.2b) with default parameters except—AlignIntronMax 1 [68]. The output files of STAR alignment (BAM format) were used as input in R-studio to produce matrices using the feature count function from the Rsubread package (v.1.34.2) [69]. The count matrices were used as input in the DESeq2 R package (v.1.24.0) for miRNA expression analysis by comparing each of the sampling groups T2, T3, T4, T5 and T6 to T1. All groups consisted of seven samples. An internal normalization is initially carried out by estimating the size factor for each sample prior to comparison of groups. The size factor is estimated by first calculating the geometric mean for each gene across all samples. The counts for a gene in each sample are then divided by this geometric mean. The median of these ratios in a sample corresponds to the size factor for that sample [70].

A threshold of log2 fold-change ≤−1.0 or ≥1.0, Benjamini-Hochberg adjusted *p*-value < 0.05 and with average normalized read counts ≥30 in at least at one comparison were used to identify DE miRNAs. Subsequently, the assumed biologically active guide DE miRNAs (gDE miRNAs) were determined by comparing the read counts of DE miRNAs originating from the same precursor. DE miRNAs that showed >10 times more reads than the other mature from the same precursor were defined as biologically active gDE miRNAs. However, in cases where two mature miRNAs from the same precursor showed a read count difference less than the threshold (10 times), both were included as putative gDE miRNAs in the enrichment analysis (4.7). All gDE miRNAs were included in the in silico target analysis and gene enrichment analysis. Finally, unsupervised hierarchical clustering with complete linkage and Spearman correlation was performed with DE-miRNA log2-fold changes as input using the hclust function from the stats package (v.3.6.1) in R. Heatmap2 from R-package gplots (v.3.0.1.1) was used to plot heatmaps of DE miRNAs grouped by the hierarchical clustering analysis.

### 4.6. Microarray Analysis of mRNA Expression

Microarray analysis was performed on 32 of the 42 gill samples chosen for HTS that represented the six time points (T1–T6), with five fish per time point. The experiment was conducted at NOFIMA (Ås, Norway) using a 44 k DNA oligonucleotide microarray containing 60-mer probes for protein coding genes (Salgeno-2, GPL28080). The probes were designed at NOFIMA and annotated with the bioinformatics package STARS [71]. The designed oligonucleotide microarrays were manufactured by Agilent Technologies (Inc., Cedar Creek, TX, USA), and the reagents and equipment used in this experiment were from the same source. One-color hybridization was used, and each of the gill samples was analyzed with a separate array.

Total RNA (220 ng) was used as input for cDNA synthesis, followed by cDNA amplification and Cy3 labeling of cRNA using the LowInput QuickAmp Labeling Kit, according to the manufacturer’s protocol. The labeled cRNAs were purified using Qiagen’s RNeasy Mini Kit (QIAGEN group, Hilden, Germany). The concentration and purity of purified cRNA were measured using a NanoDropTM1000 Spectrophometer (Nanodrop ND-1000, Thermo Fisher Scientific, Wilmington, DE, USA). The purified Cy3-labeled cRNA (1650 ng) was used as input to prepare the hybridization mix using the Gene Expression Hybridization Kit. The hybridization mix for each sample was added to each individual array and subsequently hybridized in an oven (17 h, 65 °C, rotation speed 0.01 g). After hybridization, the slides were washed and scanned with a SureScan Microarray Scanner (Agilent Technologies, Santa Clara, CA, USA). The mRNA array data were processed using Nofima’s bioinformatic package STARS [71]. The mRNA expression analysis was conducted by comparing T1 to each of the other time points (T2–T6). A threshold of log2 fold-changes ≤ –0.80 or ≥ 0.80 and *p* < 0.05 (*t*-test) was used to identify differently expressed mRNAs (DE mRNAs). Finally, enrichment analysis of DE mRNAs was carried out as described in Krasnov et al. [72] using GO, STARS and KEGG annotation data sets. Functional and pathway categories with ratio ≥1.5 and Yates’ corrected chi-square (*p* ≤ 0.05) were defined as enriched in the DE-mRNA data set.

### 4.7. DE-miRNA Target Prediction and Enrichment of Predicted Target Genes

The probe sequences of the mRNAs identified as differentially expressed in the microarray analysis were utilized to identify the full-length (FL) transcript sequences of these mRNAs in the FL transcriptome [40]. The BLASTN tool in the BLAST+ package (v.2.9.0+) [73] with settings of 90% sequence identity and 91% query coverage, was used to identify the matching FL transcripts. The set of FL transcripts identified as matches to at least one probe was used as input in the MicroSalmon GitHub repository [41] (https://github.com/AndreassenLab/MicroSalmon/, accessed on 6 May 2022) to identify the putative targets of gDE miRNAs among the DE mRNAs.

The gene symbols of FL targets needed for input in the enrichment analysis were retrieved from Microsalmon [41] and the Universal Protein Resource (UniProt) (https://beta.uniprot.org, accessed on 6 May 2022), using the FL target transcript annotation information as input. Subsequently, GO and Reactome pathway enrichment analysis was performed using the PANTHER Overrepresentation Test, which is an ORA enrichment approach (Released 2 February 2022) (http://www.pantherdb.org, accessed on 6 May 2022), GO Ontology database (Released 1 November 2020) and Reactome version 65 (Released 1 October 2021). *Homo sapiens* was used as a reference gene list in the enrichment analysis, as this is the most complete functional annotated database, while teleosts are rather incompletely annotated. A threshold of fold enrichment (FE) ≥ 2 and Fisher’s Exact test with a False Discovery Rate (FDR) less than 0.05 as calculated by the Benjamini–Hochberg procedure were used as thresholds for significance in these analyses. PANTHER grouped subclasses that were related to the same functional category or pathway category together and sorted them by most specific subclasses by default (see Supplementary Files in Section 2.5).

## Figures and Tables

**Table 1:** Expression changes of gDE miRNAs in cluster 1.

Mature miRNAs	T2 ^1^	T3 ^2^	T4 ^3^	T5 ^4^	T6 ^5^
ssa-let-7b-3p	−1.2 *	−0.1	0.2	−1.0 *	−0.6
ssa-miR-30a-1-2-5p	−0.4	−0.6	−0.6	−1.0 *	−0.9
ssa-miR-30a-3-4-5p	−0.4	−0.6	−0.6	−1.0 *	−0.9
ssa-miR-30e-5p	−1.2	−1.2	−1.4 *	−1.9 *	−1.3
ssa-miR-124abcd-3p	1.1 *	0.4	−0.1	−1.2 *	−1.5 *
ssa-miR-146a-5p	−0.5	−0.7	−0.9	−1.1 *	−0.4
ssa-miR-146b-3p	−0.6	− 1.4 *	−2.0 *	−2.4 *	−0.7
ssa-miR-146b-5p	−0.1	− 1.1 *	−1.6 *	−1.7 *	−0.4
ssa-miR-190b-5p	−1.2 *	− 1.4 *	−1.1 *	−1.7 *	−1.5 *
ssa-miR-194b-5p	−1.1 *	−1.6 *	−1.3 *	−1.1 *	−0.7
ssa-miR-1778-3p	−1.0 *	−1.6 *	−1.0 *	−0.7	−0.4
ssa-miR-7132b-5p	0.0	−0.6	−1.0 *	−0.9	−0.7
ssa-miR-7132a-5p	0.0	−0.6	−1.0 *	−0.9	−0.7

^1^ Half way through light treatment. ^2^ Three quarters into the light-treatment period. ^3^ Smolt, one day prior to SWT. ^4^ One week after SWT. ^5^ One month after SWT. All values are log2 fold-change relative to T1. The asterisk (*) indicates significant expression changes.

**Table 2:** Expression changes of gDE miRNAs in cluster 2.

Mature miRNAs	T2 ^1^	T3 ^2^	T4 ^3^	T5 ^4^	T6 ^5^
ssa-miR-101a-3p	1.1 *	0.6	0.7	0.7	0.4
ssa-miR-193b-5p	0.2	0.5	0.7	1.1 *	1.4 *
ssa-miR-499a-5p	0.7	0.7	1.0 *	0.6	0.8
ssa-miR-727a-3p	0.6	0.9	0.8	1.2 *	0.9
ssa-miR-8159-5p	0.4	0.7	0.6	0.8	1.0 *

^1^ Half way through light treatment. ^2^ Three quarters into the light-treatment period. ^3^ Smolt, one day prior to SWT. ^4^ One week after SWT. ^5^ One month after SWT. All values are log2 fold-change relative to T1. The asterisk (*) indicates significant expression changes.

**Table 3:** gDE miRNAs targeting *nkaα1a*, *nkaβ* and *cyp1a1* isoforms.

Gene	FL Isoforms /Paralog Transcript Accession	gDE miRNAs
*nkaα1a*	GIYK01009408; GIYK01019309; GIYK01009409	ssa-miR-8159-5p
	GIYK01019303	ssa-miR-499a-5p
*nkaβ*	GIYK01043074; GIYK01043155; GIYK01043071; GIYK01043154; GIYK01043075; GIYK01043153; GIYK01043151; GIYK01043150	ssa-miR-146b-5p ssa-miR-146a-5p ssa-miR-1778-3p
*cyp1a1*	GIYK01057368; GIYK01027094; GIYK01057370; GIYK01027092; GIYK01027093; GIYK01057371; GIYK01057369	ssa-miR-1778-3p

**Table 4:** Enriched gene pathways.

Pathway Category	Reactome Pathways	FE ^1^	FDR ^2^
Extracellular matrix org.	Integrin cell surface interactions	4.3	5.0E-03
Cytokine signaling	Interferon Signaling	2.9	1.0E-02
	Cytokine Signaling in Immune system	2.3	2.0E-05
	Signaling by Interleukins	2.4	4.5E-04
Innate immune system	Neutrophil degranulation	2.2	4.6E-03
	Innate Immune System	1.9	9.8E-05
Adaptive immune system	Adaptive Immune System	1.7	3.4E-02
Hemostasis	Cell surface interactions at the vascular wall	2.7	2.4E-02
	Platelet activation, signaling and aggregation	2.7	4.1E-03
Metabolism	Metabolism of vitamins and cofactors	2.7	3.2E-02
	Metabolism of lipids	2.2	3.9E-05
Signal transduction	Signaling by Receptor Tyrosine Kinases	2.4	1.1E-04
Disease	Diseases of signal transduction by growth factor receptors and second messengers	2.2	1.6E-02

^1^ The Fold Enrichment of the genes observed in the analyzed list over the expected. Fold enrichment is generated by dividing the number of genes in the analyzed list by the expected number. If it is greater than 1, it indicates that the category is overrepresented in the analyzed list. ^2^ The False Discovery Rate as calculated by the Benjamin-Hochberg procedure.

**Table 5:** gDE miRNAs in the gill and their expression status in similar studies of the liver and HK.

gDE miRNAs in Gill	DE miRNAs in Liver ^1^	DE miRNAs in Head Kidney ^2^
ssa-let-7b-3p	Yes	Yes
ssa-miR-146a-5p	Yes	Yes
ssa-miR-146b-5p	Yes	Yes
ssa-miR-30a-1-2-5p	Yes	No
ssa-miR-30a-3-4-5p	Yes	No
ssa-miR-30e-5p	Yes	No
ssa-miR-101a-3p	Yes	No
ssa-miR-194b-5p	No	Yes
ssa-miR-124abcd-3p	No	No
ssa-miR-146b-3p	No	No
ssa-miR-190b-5p	No	No
ssa-miR-193b-5p	No	No
ssa-miR-499a-5p	No	No
ssa-miR-727a-3p	No	No
ssa-miR-1778-3p	No	No
ssa-miR-7132a-5p	No	No
ssa-miR-7132b-5p	No	No
ssa-miR-8159-5p	No	No

^1^ Results from Shwe et al. [49]. ^2^ Results from Shwe et al. [50].

**Table 6:** Photoperiod and water temperature during the experimental trial.

Experimental Days	Hours of Light per Day (h)	Water Temperature (°C)	Water Type
Day 0	24	8	Fresh water
Day 1–5	12	13	Fresh water
Day 6–47	12	12	Fresh water
Day 48–60	24	12	Fresh water
Day 61–81	24	8	Fresh water
Day 82–111	24	8	seawater

**Table 7:** Time points and conditions where gill samples were collected.

Group	Sample Collection Time Points	L ^1^	T ^2^	Weight ^3^	Water Type	Samp ^4^
T1	Parr, one day prior to light treatment	24	8	29.4 ± 5.6	Fresh water	Day 0
T2	Halfway into light treatment	12	12	52.6 ± 5.9	Fresh water	Day 47
T3	Three quarters into light treatment	24	8	63.9 ± 10.1	Fresh water	Day 67
T4	Smolt, one day prior to SWT	24	8	72.4 ± 8.7	Fresh water	Day 81
T5	One week after SWT	24	8	63.2 ± 8.5	seawater	Day 88
T6	One month after SWT	24	8	98.4 ± 14.9	seawater	Day 111

^1^ Hours with day light. ^2^ Water temperature in degrees Celsius (°C). ^3^ Average weight in grams of the experimental fish collected at each time point. ^4^ Sampling day within the experimental period.

## Data Availability

All sequenced samples have been submitted to the NCBI Sequence Read Archive Centre (SRA) (https://www.ncbi.nlm.nih.gov/sra, accessed on 3 June 2022) with accession bioproject number PRJNA681317.

## Supplementary Materials

The following supporting information can be downloaded at: https://www.mdpi.com/article/10.3390/ijms23158831/s1.
