# Supplementary material for: Differential Expression of miRNAs and Their Predicted Target Genes Indicates That Gene Expression in Atlantic Salmon Gill Is Post-Transcriptionally Regulated by miRNAs in the Parr-Smolt Transformation and Adaptation to Sea Water
Source: Int J Mol Sci. 2022 Aug 8;23(15):8831. doi: 10.3390/ijms23158831 (PMC9369087; doi:10.3390/ijms23158831)
Supplement: Supplementary file 1 [file ijms-23-08831-s001.zip › Figure S1. Heatmap of 32 DE-miRNAs.pdf]

Heatmap and hierarchical clustering of the 32 differentially expressed miRNAs in gill of Atlantic salmon

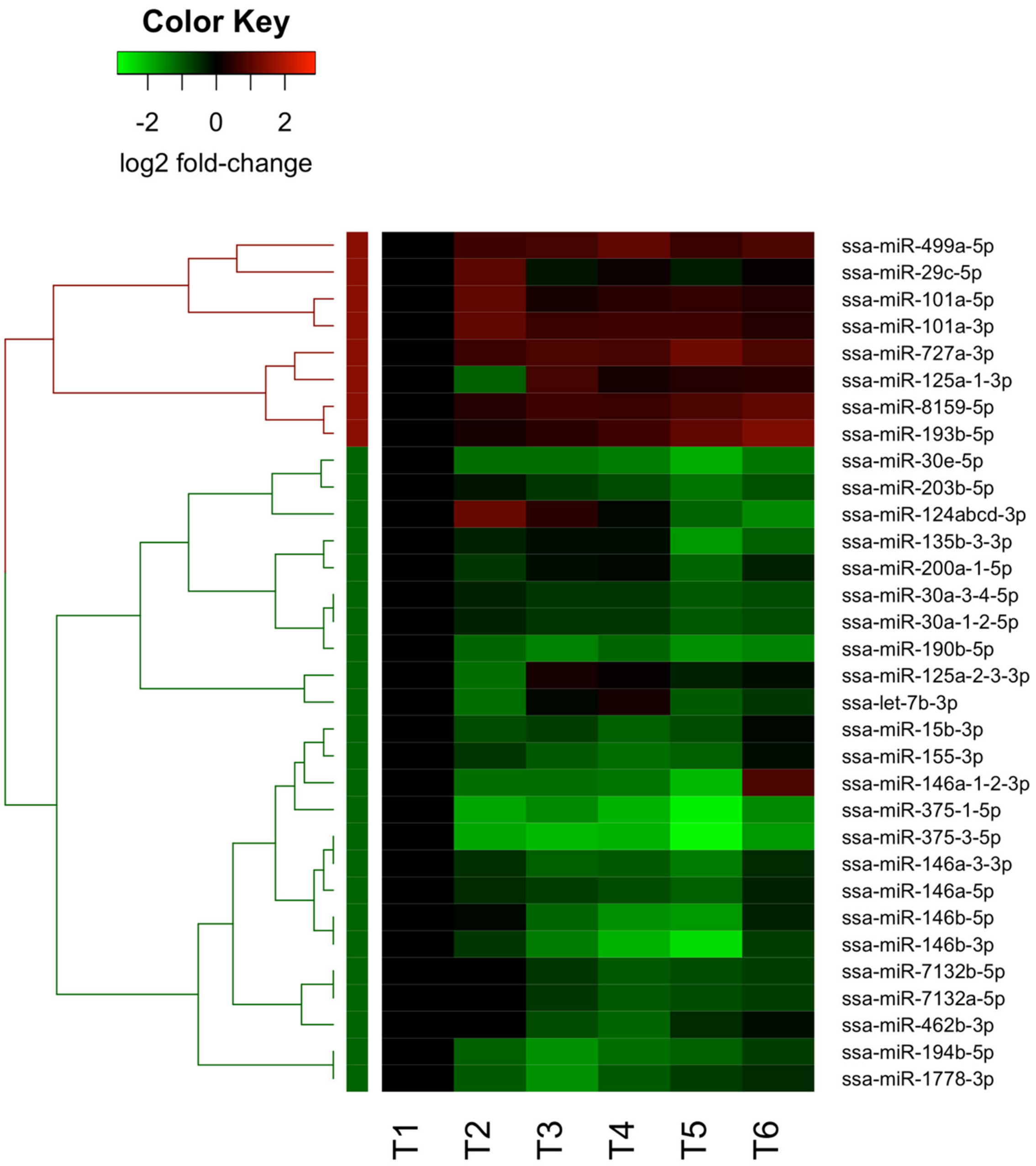

Figure S1. Heatmap and hierarchical clustering of the 32 differentially expressed miRNAs (DE-miRNAs). Each row represents a miRNA and each columns represents the expression changes at each time points relative to T1 (pre-smolt, one day before smoltification). T2-T4 and T5-T6 are relative expression changes during smoltification period and post SWT period, respectively. The dendrogram and the row side colours on the left show the two major clusters of DE-miRNAs (Cluster 1 – red and Cluster 2 – green).
